# Barriers and facilitators to infection control at a hospital in northern India: a qualitative study

**DOI:** 10.1186/s13756-017-0189-9

**Published:** 2017-04-08

**Authors:** Anna K. Barker, Kelli Brown, Dawd Siraj, Muneeb Ahsan, Sharmila Sengupta, Nasia Safdar

**Affiliations:** 1 Department of Population Health Sciences, University of Wisconsin-Madison, School of Medicine and Public Health, Madison, WI USA; 2 Department of Medicine, University of Wisconsin-Madison, School of Medicine and Public Health, Madison, WI USA; 3 Medanta Institute of Eduation and Research, Medanta the Medicity Hospital, Gurgaon, Haryana India; 4 Department of Clinical Microbiology & Infection Control, Medanta the Medicity Hospital, Gurgaon, Haryana India; 5 William S. Middleton Memorial Veterans Affairs Hospital, Madison, WI USA

**Keywords:** Infection control, Global health, Qualitative methodology, Human factors, India

## Abstract

**Background:**

Hospital acquired infections occur at higher rates in low- and middle-income countries, like India, than in high-income countries. Effective implementation of infection control practices is crucial to reducing the transmission of hospital acquired infections at hospitals worldwide. Yet, no comprehensive assessments of the barriers to sustained, successful implementation of hospital interventions have been performed in Indian healthcare settings to date. The Systems Engineering Initiative for Patient Safety (SEIPS) model examines problems through the lens of interactions between people and systems. It is a natural fit for investigating the behavioral and systematic components of infection control practices.

**Methods:**

We conducted a qualitative study to assess the facilitators and barriers to infection control practices at a 1250 bed tertiary care hospital in Haryana, northern India. Twenty semi-structured interviews of nurses and physicians, selected by convenience sampling, were conducted in English using an interview guide based on the SEIPS model. All interview data was subsequently transcribed and coded for themes.

**Results:**

Person, task, and organizational level factors were the primary barriers and facilitators to infection control at this hospital. Major barriers included a high rate of nursing staff turnover, time spent training new staff, limitations in language competency, and heavy clinical workloads. A well developed infection control team and an institutional climate that prioritizes infection control were major facilitators.

**Conclusions:**

Institutional support is critical to the effective implementation of infection control practices. Prioritizing resources to recruit and retain trained, experienced nursing staff is also essential.

**Electronic supplementary material:**

The online version of this article (doi:10.1186/s13756-017-0189-9) contains supplementary material, which is available to authorized users.

## Background

Healthcare associated infections (HAIs) affect millions of patients every year and are the most common complication of healthcare delivery globally [1]. They complicate clinical care, increase length of hospital stays, and are particularly debilitating for patients and healthcare facilities with limited income and resources [2–4].

While HAIs are a considerable problem in high-income countries, low- and middle-income counties are disproportionately burdened by these infections [5, 6]. In India, a majority of healthcare settings lack robust infection control infrastructure and no nationwide HAI surveillance system exists [7]. Low- and middle-income countries also tend to have higher rates of antimicrobial resistance [5]. A recent multi-center study conducted at twelve Indian intensive care units found an overall rate of 9.06 HAIs per 1000 intensive care days, which is close to the global average in high income countries [8]. However, there is considerable variability in infection rates at institutions across the country. Several single site studies have reported considerably higher HAI rates, with levels reaching between 25 and 40 infections per 1000 patient days [9–11].

Effective implementation of infection control practices is crucial to controlling the transmission of HAIs in settings with high infection rates. A recent meta-analysis found that over ninety-five percent intervention compliance is required to reduce central line-associated bloodstream infections [12]. While the necessary rate of compliance is not known for other infections, all infection control interventions are complex, multifaceted, and challenging to sustain [13]. In order for infection prevention measures to be successful, barriers to effective implementation must be identified and overcome [14]. Likewise, facilitators to intervention implementation must also be identified and championed.

**Fig. 1:**
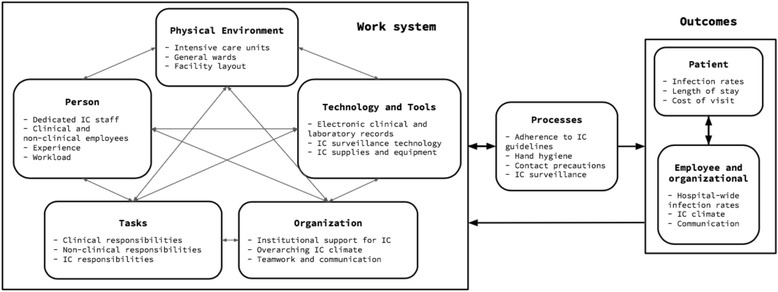
SEIPS model of infection control in an Indian hospital. Adaptation of the SEIPS model by Carayon, et al. to infection control in an Indian hospital [15]. The work system includes five factors: tools and technology, organization, environment, person, and tasks. These affect related processes and outcomes. IC: infection control

The Systems Engineering Initiative for Patient Safety (SEIPS) is one of the leading conceptual frameworks in human factors engineering research [15]. This model examines problems through the lens of complex interactions between people and systems, which includes organizations, technology and tools, the environment, tasks, and people (Fig. 1). It has been utilized as the guiding framework for patient safety analyses in over fifty studies, ranging from primary care clinics to intensive care units (ICUs [16]). The SEIPS model improves upon earlier patient safety frameworks by evaluating both the causes and control of medical errors [15]. Thus, it is a natural fit for investigating the behavioral and systematic components of infection control practices. SEIPS has previously been used to identify barriers and facilitators to infection prevention practices for *Clostridium difficile* infection [17], ventilator associated pneumonia [18], and HAIs in the ICU [19].

Despite the high rate of HAIs in India, no study of the comprehensive barriers and facilitators to infection control has been conducted at an Indian hospital, to our knowledge. Thus, we conducted a qualitative study of infection control practices at a tertiary care hospital in Haryana, northern India, based on the SEIPS conceptual framework.

## Methods

We conducted a qualitative study of facilitators and barriers to infection control at a private tertiary care hospital in Haryana, India. Semi-structured interviews were conducted in June and July 2015.

### Study population

Ten physicians and ten nurses were recruited at a 1250-bed tertiary care private hospital in Haryana, India. The hospital includes ten ICUs for a total of 350 ICU beds. Participants were selected by convenience sampling and represented a wide range of clinical departments and career levels. They were recruited in the hospital employee cafeteria, on clinical wards, and by word of mouth. To ensure a range of clinical expertise, some participants from less common sectors, for example infectious disease nursing and ICU leadership, were approached directly. All hospital employees directly involved in patient care were eligible for enrollment. Student trainees and non-English speakers were excluded, although no potential participants met exclusion criteria.

### Interviews

We conducted twenty semi-structured interviews to assess facilitators and barriers to a hospital infection control program. English language competency is a requirement for employment as a healthcare worker at the study hospital, thus, all interviews were conducted in English. Interviews took place at the hospital, in a room adjacent to the participant’s work environment. The initial interview guide was developed based on the SEIPS model (Fig. 1) and was refined during the study based on participant responses (Additional file 1). Interview questions assessed the hospital’s infection control policies, focusing on how people, physical environments, tasks, organizations, and tools are barriers and facilitators to the success of ongoing interventions. Most interviews lasted between ten and twenty minutes. All were audio recorded and transcribed for qualitative analysis.

### Analysis

Preliminary data analyses were conducted concurrently with study procedures to direct iterative revisions of the interview guide and determine theoretical saturation. Reworking the interview guide allowed the focus of the interview content to shift overtime, so that new information was gleaned even among later participants. After interviewing the twentieth participant, we decided that the responses to interview questions were becoming highly repetitive and that no new data were likely to appear. Thus, a twenty participant sample size was finalized based on theoretical saturation. Interview transcripts were subsequently analyzed using NVivo software (Version 11.3, QSR International), with responses coded for themes based on the SEIPS framework [15]. NVivo is a qualitative software that aids data analysis by organizing the key themes that are identified by researchers in large quantities of open-ended text. Data were independently coded in NVivo by two investigators (AB and KB) who then jointly reviewed the analyses.

### Ethics approval

The institutional review board of the participating institution approved this study and the Health Sciences Institutional Review Board at the University of Wisconsin- Madison granted this study exemption from review. All participants provided oral consent before any data were collected.

## Results

### Participants’ characteristics

Participants were comprised of ten nurses (80% female) and ten doctors (30% female), selected from a wide range of clinical departments including internal medicine, neurology, anesthesia, infectious disease, and three ICUs (cardiac, pediatric, and medicine). Three nurses were recruited from the hospital’s infection control department. Participants were enrolled from a range of career levels including junior and senior consulting physicians, residents, head nurses, and general nursing staff.

### Tools and technology

Hospital staff overwhelmingly reported that an adequate supply of contact precautions equipment was readily available for use (Table 1). On the general wards, gowns, gloves, and masks were stored outside of the rooms of patients placed under contact precautions. Gloves, masks, and shoe covers, for dust control, were available within and outside of the ICUs, with gowns available inside the rooms of patients under contact precautions. Although mask use was only required when interacting with patients under droplet or airborne precautions, healthcare workers often put on a mask before entering the ICU. They wore masks intermittently in the ICU and switched them after visiting patients under infection control precautions. Despite their availability, participants reported that both healthcare workers and visitors struggled with mask compliance for patients under droplet or airborne precautions, in large part because of issues surrounding comfort. *“We have to remind visitors all the time [to wear the mask…] The mask is very difficult, especially when they are in the room. If they are sitting there all the time, the mask makes them very hot.” -Internal medicine resident;* “*Staff know the importance of personal protective equipment, but they do not always wear the mask. […] They report that when they wear the mask for a long time, they feel like they are suffocating.” -Infection control head nurse.*

**Table 1:** Barriers and facilitators to infection control, categorized by components of the Systems Engineering Initiative for Patient Safety (SEIPS)

Tools and Technology	Organization	Environment	Person	Tasks
Barriers				
Clinician and visitors find mask uncomfortable	High nursing turnover	Shoe covers required in ICUs because of dust	New nurse hires often lack clinical experience	Heavy patient workload
No comprehensive electronic health record	Limited initial Hindi language capabilities	Ongoing construction within hospital	Frustration with the frequency of IC training	Perceived understaffing
Facilitators				
Ample IC supplies	Institutional climate that prioritizes IC	Centrally located sinks, hand gel at bedside	IC team well integrated into clinical care	Staff knowledgeable about IC practices
Electronic database for laboratory results	Funding and support for dedicated IC team	Sufficient beds to prevent overcrowding	Large environmental cleaning staff (600)	IC nurses help new hires complete IC tasks

*IC* infection control, *ICU* intensive care unit

Although the hospital’s health record was not fully electronic, an electronic microbial database served as the primary resource for treating physicians to access final laboratory results. Critical results were communicated from the microbiologist to the physician by phone and documented. Infection control nurses also utilized the electronic database for tracking patients, accessing microbiology results, and ensuring that correct contact precautions signage was promptly hung and precautions implemented.

### Organization

Physicians identified nursing staff turnover as the single greatest barrier to infection control. This was also identified as a barrier by the infection control nurses, but not general nursing staff. Several participants reported high turnover rates, with nurses leaving primarily to take higher paying positions abroad. This was complicated by the fact that many new nurses entered directly from nursing school, without any prior work experience. *“We have a huge turnover of nurses in our hospital. We lose around one-third of our intensive care staff every six months to the west or the Middle East.” -ICU, senior physician.* While physicians knew that investing in the training of new nurses was crucial to patient care, it was also acknowledged to be time consuming and rarely resulted in long-term benefits for the healthcare team.

Participants also identified the limited Hindi language capabilities of incoming nurses as a considerable barrier to effective infection control. This was cited as a concern by general and infection control nurses, but no physicians. Hindi was the primary language used between healthcare workers when discussing clinical care. It is predominately spoken in northern India, but a majority of the nursing schools nationwide are located in the south. Thus, many nurses join the hospital with limited Hindi speaking abilities and initially struggle to communicate with patients and other staff. *“There is a language barrier because a majority of nurses are from South India, but here (in Haryana), they use Hindi. […] In two or three months the nurses will pick up the language. Before, when I lived in southern India, I did not know Hindi. Now I do.” -Staff nurse.*

Because most infection control training takes place in the first few months of employment, this is particularly hindered by limited language proficiency. “*The nurses from Kerala [a southern state of India] do not know much Hindi or English, so sometimes we wonder how we should teach them to make them understand. […] There are so many things that are very difficult to teach them.” Infection control staff nurse.* To help new nurses learn Hindi, head nurses often communicated with them in broken Hindi, even if they shared the same local language from the south.

Despite these barriers, the organization was committed to infection control and has facilitated it through the creation and staffing of a large infection control team. This includes sixteen nurses dedicated full-time to infection control activities. The hospital has also designated several physicians as leaders in ongoing infection control and antibiotic stewardship initiatives. These actions have created an institutional climate that prioritizes and values infection control. *“Other institutions talk about infection control, but nobody emphasizes it like they are doing now. I have realized that this [hospital-acquired infections] is a major reason why my patients are staying in the hospital longer.”-Anesthesia, physician.*

### Environment

The hospital’s physical environment was structured and maintained to facilitate infection control. Each general ward had a centrally located sink with running water and alcohol based hand rub at the patients’ bedside. Rooms contained between one and six beds and patients under contact precautions were either placed into single rooms or cohorted. Overcrowding was not a problem and every patient was given their own bed. Each ICU had several sinks and a hand rub bottle for each patient.

Environmental cleaning was prioritized by the hospital, which employs more than six hundred cleaning staff on site. Housekeeping staff were recognized by study participants as key stakeholders in infection prevention. Anecdotally, they had the best hand hygiene compliance rates in the hospital and participants were adamant that environmental cleaning was comprehensive and timely. Staff decontaminated patient rooms three times a day, with common areas cleaned even more frequently. *“Every two to three hours [housekeeping] will come.”-Cardiac ICU, senior staff nurse.*

### Person

Sixteen nurses were dedicated to front-line implementation of hospital infection control policies. Each worked on specific units rounding with physicians, conducting daily hand hygiene and environmental cleaning audits, and providing new nurses with infection control training at the patient’s bedside. Because of the high rate of nursing turnover, these trainings occurred on a daily basis. *“I am very strict and will say to them [the nurses] again and again, don’t do this or don’t do that. I instruct them, because I do not want my staff transmitting infections from one patient to another.” - Infection control staff nurse.*

Infection control nurses were also responsible for ensuring that nursing staff completed infection control checklists for high risk patients at each shift. Most nurse and physician participants were receptive to the work of the infection control nurses. *“If the nurse sees that someone is not doing it [hand hygiene], she will point it out, whether it is a doctor or a nurse. She’s like a police woman. […] We always do whatever she says, because even we forget that infection is such a problem in the ICUs. We have to take advice from her. We do not mind.” -ICU, senior physician.* A few non-infection control nurses described frustration with the continual training and audits. *“If it is a new person then it is fine, because they need training. But if you’ve been here for awhile, it is not so good.” -Staff nurse.*

### Tasks

The typical nurse to patient ratio on the general wards was 1:6 and 1:1 or 1:2 in the ICU. Several non-infection control nurses expressed concern that they were understaffed and the daily workload was difficult to manage. *“We are not getting any time to sit, or even to stand. We have to run just like in the Olympics.” -Pediatrics ICU, staff nurse.*

The perceived workload had direct implications for infection control practices. Both staff nurses and infection control nurses reported that staff nurses were less likely to practice infection control properly when they were busy. *“The knowledge is there, but some people are not implementing it [the practices…] If they are busy, sometimes they avoid it.” -Infection control nurse.*

## Discussion

In this interview-based, qualitative analysis of the barriers and facilitators to infection control implementation in an Indian hospital, we found that staff turnover, time spent training new staff, limitations in language competency, and workload were major barriers to effective infection control. A well developed infection control team and an institutional climate that prioritizes infection control were major facilitators. Most of these barriers and facilitators mapped to the tasks, person, and organizational components of SEIPS.

These findings have implications for hospital leadership, infection control departments, and clinicians. It is especially imperative in resource constrained environments to implement interventions that are the most likely to be impactful. With the recognition of major barriers related to staffing, support from the highest levels of leadership is needed to implement policies that incentivize healthcare worker retention and recruitment. Hospital leadership must also prioritize the allocation of resources to rapidly build language and infection control skills for incoming healthcare workers. We recommend that hospitals incorporate intensive language training focused on medically relevant vocabulary into initial staff onboarding programs.

Two interventional studies from India also report on the role of person and organizational level factors in improving the implementation of infection control interventions. The first, assessing airborne infection control practices in thirty-five Indian healthcare settings, found that an intervention bundle focused on capacity building and systems development at the organizational level substantially improved the implementation of infection control policies [20]. The second study introduced an organizational change process intervention known as appreciative inquiry to the maternity wards of three hospitals in Gujarat, India. Researchers found that the introduction of the appreciative inquiry program improved decision making and inter-personal relationships between healthcare workers, which in turn facilitated improved implementation and compliance of infection control practices [21]. Furthermore, a recent international survey of infection prevention practices in thirty countries found that limited trained staff, infrastructure, and supplies were major barriers to preventing multidrug resistant organism transmission [22].

In our study, responses regarding the SEIPS factors physical environment and tools and technology primarily discussed facilitators to infection control. Participants reported an ample supply of gowns, masks, and gloves, placed in highly visible locations. Barriers to compliance centered around complex behavioral issues, instead of a lack of supplies. In contrast, a lack of personal protective equipment has previously been identified as a barrier to infection control in other Indian hospitals [21, 23]. These findings must be contextualized for the study institutions. One limitation of our study is that it focuses on a single tertiary care hospital that is internationally accredited by Joint Commission International. As such, our findings are likely not generalizable to India’s more resource limited healthcare settings.

Additional studies are needed from public government hospitals and other institution types. The Indian healthcare system is varied and complex with a high burden of antimicrobial resistance. Organized infection control programs are not routinely practiced, except in accredited healthcare organizations. Data regarding the barriers and facilitators from multiple types of healthcare settings are required to create a broader action plan of curbing antimicrobial resistance and healthcare associated infections.

Another limitation of the study is that we were not able to correlate self-reported practices with direct observations of infection control behavior. Future studies may identify additional barriers and facilitators of relevance by collecting such data via direct observation of hospital infection control practices.

Despite these limitations, we conducted, to our knowledge, the first SEIPS work system analysis of infection control practices at a hospital in India. Furthermore, our findings and methods provide institutions with an approach to identifying local barriers and facilitators to infection prevention that guide intervention implementation. Adaptation and tailoring interventions to local contexts is crucial. Designing solutions without identifying and accounting for such underlying inter-related barriers may lead to unsuccessful interventions and is a potential reason why interventions that have been successful in one setting may fail in another.

## Conclusions

A work systems evaluation is a valuable exercise for organizations to identify new and evolving areas for improvement. At our Indian study hospital, tasks, person, and organizational level factors were key to the success of infection control practices. Institutional support for infection control and prioritizing resources to recruit and retain trained, experienced nursing staff are critical to the effective implementation of infection control practices.

## Additional file

Additional file 1: Initial semi-structured ﻿interview guide, based on the SEIPS framework. (PDF 139 kb)

## Abbreviations

HAI: Healthcare associated infections
ICU: Intensive care unit
SEIPS: Systems engineering initiative for patient safety
