# Supplementary material for: Barriers and facilitators to infection control at a hospital in northern India: a qualitative study
Source: Antimicrob Resist Infect Control. 2017 Apr 8;6:35. doi: 10.1186/s13756-017-0189-9 (PMC5385016; doi:10.1186/s13756-017-0189-9)
Supplement: Additional file 1: — Initial semi-structured ﻿interview guide, based on the SEIPS framework. (PDF 139 kb) [file 13756_2017_189_MOESM1_ESM.pdf]

An initial interview guide has been developed based on the systems engineering initiative for patient safety (SEIPS) framework described in the protocol, and will be frequently reworked based on participant responses.

Pre-constructed questions to direct the interview include:

Person:
What is your job at the hospital? How long have you worked at this job? Have you ever worked for a hospital infection control department before?
For physician participant (P): Approximately how many patients do you see in a day?
For nurse participant (N): What is the approximate nurse:patient ratio on your ward?

Tasks:
What practices are done on your ward to prevent infections (ex: contact isolation, hand hygiene, etc.)? What would make it easier to do these practices?
P: Are *Clostridium difficile* (*C. difficile*) infections a concern on your ward? How frequently do you order *C. difficile* stool testing?
P: Would conducting routine surveillance of *C. difficile* or other antibiotic resistant infections (MRSA, VRE, etc.) be helpful or unnecessary on your ward?
N: What infection control practices are you responsible for on the ward?

Tools:
How are patients with antibiotic resistant infections identified 1) in the health record, and 2) at the bedside?
Are gowns, gloves, and masks available when needed? Explain.
P: What is the microbiology lab capacity for antibiotic resistance testing? Is it adequate for your clinical needs?
P: How is the diagnosis of antibiotic resistance from an outside hospital used on admission for ordering isolation precautions and confirmatory laboratory testing?

Organization:
Do staff receive any ongoing education regarding proper infection control practices?
Is staff feedback asked for regarding new infection control policies?
What makes it difficult to implement new infection control policies on your ward?
What helps to facilitate communication between the different types of healthcare workers on your ward (doctors, nurses, etc.)?
P: How soon are positive antibiotic resistant results reported to you? How are they reported (electronic alert, phone, in person, etc.)?
P: Are you aware of the new antibiotic stewardship policy that was implemented at the hospital in April 2015? How has this impacted your clinical practice?

Environment:
What types of environmental decontamination is conducted on your ward? How frequently do they occur?
Where are the sinks located on your ward? Where is hand sanitizer gel located?
Where can patients wash/gel their hands on your ward?
